# Error in Conflict of Interest Disclosures

**DOI:** 10.1001/jamanetworkopen.2025.41219

**Published:** 2025-10-08

In the Consensus Statement titled “Proposed Nutrition Competencies for Medical Students and Physician Trainees: A Consensus Statement,” published on September 30, 2024, the Conflict of Interest Disclosures for Dr Maile incorrectly stated that he reported receiving personal fees from Nestle Professional. This article has been corrected online.^1^
